# Testing Variational Bias Correction of Satellite Radiance Data in the ACCESS-C: Australian Convective-Scale NWP System

**DOI:** 10.3390/s22239504

**Published:** 2022-12-05

**Authors:** Nahidul Hoque Samrat, Fiona Smith, Jin Lee, Andrew Smith

**Affiliations:** The Bureau of Meteorology, Melbourne, VIC 3001, Australia

**Keywords:** data assimilation, bias, numerical weather prediction, regional models, satellite radiance

## Abstract

Radiance observations are typically affected by biases that come mainly from instrument error (scanning or calibration) and inaccuracies of the radiative transfer model. These biases need to be removed for successful assimilation, so a bias correction scheme is crucial in the Numerical Weather Prediction (NWP) system. Today, most NWP centres, including the Bureau of Meteorology (hereafter, “the Bureau”), correct the biases through variational bias correction (VarBC) schemes, which were originally developed for global models. However, there are difficulties in estimating the biases in a limited-area model (LAM) domain. As a result, the Bureau’s regional NWP system, ACCESS-C (Australian Community Climate and Earth System Simulator-City), uses variational bias coefficients obtained directly from its global NWP system ACCESS-G (Global). This study investigates independent radiance bias correction in the data assimilation system for ACCESS-C. We assessed the impact of using independent bias correction for the LAM compared with the operational bias coefficients derived in ACCESS-G between February and April 2020. The results from our experiment show no significant difference between the control and test, suggesting a neutral impact on the forecast. Our findings point out that the VarBC-LAM strategy should be further explored with different settings of predictors and adaptivity for a more extended period and over additional domains.

## 1. Introduction

Satellite radiance observations are critical for a good forecast performance, especially where conventional observations are limited (e.g., in the Southern Hemisphere). Many numerical weather prediction (NWP) centres have used these radiance observations as input over the last few decades to help determine the most accurate initial and boundary conditions for NWP models through the variational data assimilation approach, usually referred to as 3D or 4D-Var (3- or 4-dimensional variational assimilation) (e.g., [1,2]). However, statistics from data assimilation show there are systematic differences between observations and the equivalent simulated values computed from model fields via a fast radiative transfer (RT) model. Whilst the model itself has biases, part of the difference is also attributable to the observations. Observation biases or systematic errors come from any of (but more usually a combination of) the following sources: instrument error scanning or calibration), radiative transfer error (spectroscopy or RT model), and cloud/rain/aerosol screening errors (e.g., residual clouds and aerosols) [3]. If the observations or model background contains biases, the radiance observations are not used in data assimilation (DA) systems optimally. Dee and da Silva [4] explored the removal of model bias during data assimilation; whilst model bias correction is not implemented at most forecast centres, bias correction to remove the systematic errors attributable to observations is one of the essential steps in DA systems. The purpose of this bias correction is to remove as little of the bias attributable to the model as possible; hence, the goal is not a net-zero bias for each observation type.

Satellite radiance observation biases are typically removed before or during the assimilation using parametric models (e.g., [5]). The method of bias correction is similar, regardless of whether 3D-Var or 4D-Var is used. In principle, satellite radiance bias correction methods can be classified into either static or variational (VarBC) schemes [6,7]. Both schemes typically involve a linear prediction model that calculates the bias using a set of model and observation geometry predictors. In both static and VarBC schemes, each bias is estimated with weighted predefined predictors that describe the bias at each assimilation cycle. These predictors mostly vary depending on observation characteristics (e.g., solar elevation and satellite scan angle) and atmospheric characteristics (the thickness of a particular atmospheric layer, skin temperature, etc.), but constant terms can also be incorporated. The main difference between VarBC and static bias correction is that, in static schemes, the weights given to each predictor are calculated offline from days to months of observation model fit statistics, whereas, in VarBC, the predictors form part of the control vector of the DA scheme, and the weights for each predictor are updated at each DA cycle. Today, most centres use VarBC schemes (e.g., [8,9]), and VarBC is also operational at the Bureau of Meteorology (the Bureau) in the global model. It is very important for any bias correction scheme that uses model values as predictors (such as atmospheric thickness, or skin temperature) that the model also assimilates plenty of observations that are near to unbiased to ensure the model itself is as bias free as possible. These unbiased observations, which may be Global Navigation Satellite System Radio Occultation (GNSS-RO), from aircraft or radiosonde profiles are known as “anchor” observations.

Although VarBC is widely used in global DA systems, it is yet to be fully established in regional DA because of difficulties in estimating biases in the limited-area domain [10]. A survey carried out by the International (A)TOVS Working Group, the main international scientific working group for satellite sounder data, identified that the main difficulties for implementing satellite radiance VarBC in limited-area models (LAMs) are: (1) the limited number of sounder observations, (2) nonuniform data sampling resulting in highly variable patterns of usage at different analysis times (for polar-orbiting satellites), and (3) a limited number of unbiased anchor observations. Many NWP centres tackle these difficulties in LAMs by adopting the bias coefficients from their global model (e.g., Météo-France, Japan Meteorological Agency, Canadian Meteorological Centre, and Bureau of Meteorology) [10]. These centres assume that: (1) the same set of VarBC predictors and the same radiative transfer model is used in global model and LAM, and (2) the differences between radiance biases in global and regional models are not significant. However, biases in global and regional systems can disagree due to differing model complexities, e.g., differences in the height of the top of the model (LAMs usually stop at around 40 km), specific features of the model domain, different model physics, or a significant difference in the horizontal resolution.

Consequently, Randriamampianina et al. [3] investigated the different bias correction methods in a LAM using Advanced Microwave Sounding Unit-B (AMSU-B) channels. They suggested that the use of the global bias correction coefficients showed inconsistent impacts on short-range forecasts, especially in the lower troposphere. However, LAM bias correction coefficients provided a “stable” effect on the analysis and the short-range forecasts. Recently, Benáček et al. in [10] used polar-orbiting satellite instruments (e.g., ATOVS) and assessed various VarBC configurations in the limited-area domain, including a 1.5-month initialization and 2-month evaluation period. Their extensive test shows that the VarBC-LAM methods outperform the use of VarBC coefficients from the global model (VarBC-global) in terms of stability. There was, however, little difference in forecast impact at 24 and 48 h. In both studies, the authors used a 3D-Var DA scheme.

Few centres have published experiments to justify their choices in the use of VarBC in regional models. The majority of the published work comes from Scandinavia (e.g., [3]), where there is a high degree of satellite radiance coverage from Polar orbiters and a larger number of upper air anchor observations than is typical for Australian domains. The present study has therefore been performed to better understand the impact of implementing VarBC for satellite radiances in an Australian limited-area domain using a 4D-Var DA scheme and aims to document our experiments as a starting point for further studies. The assimilation experiment incorporated satellite radiance data into a regional convective scale model over a period of nearly two months, from February 2020 to April 2020, and compared the performance of a VarBC scheme run in this configuration against the use of VarBC weights from the Bureau global NWP System.

## 2. System Configuration and Methods

### 2.1. Data Assimilation System

DA is a mechanism for combining observations with a model forecast to infer knowledge about the current state of the Earth system (e.g., atmosphere, land, and sea). 4D-Var is considered the most successful method for NWP systems. Most major NWP centres (e.g., the Bureau, Met Office, ECMWF, Météo-France, Canada, and Japan) have been using this method for their global weather forecasting for many years (e.g., [1,11,12,13]), and most have implemented it in their LAMs as well.

The assimilation system described in this study is equivalent to the current Bureau operational Australian Community Climate and Earth System Simulator—City ACCESS-C system [14,15]. It is a 4D-Var system that is based on the Met Office atmospheric model and is used operationally in real time at the Bureau. ACCESS-C uses the Met Office unified model (UM) and the associated 4D-Var analysis system (VAR), observation processing system (OPS), and the surface fields processing system (SURF).

**Figure 1:**
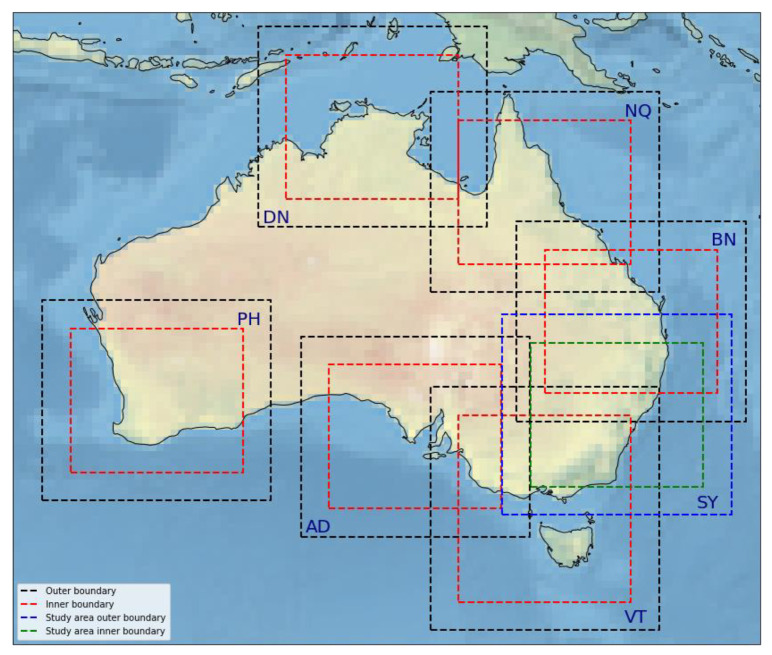
The seven Australian convective-scale variable resolution domains of the Bureau NWP System with the inner and outer grid boundaries (black and red colour). The boundary of the study area (Sydney domain) is shown in different colours (blue and green).

ACCESS-C runs with a 1-h cycle 24 times per day (0000 UTC, 0100 UTC, 0200 UTC, 0300 UTC, etc.) over seven domains: Perth (PH), Adelaide (AD), Victoria-Tasmania (VT), Sydney (SY), Brisbane (BN), Darwin (DN), and North Queensland (NQ), as shown in Figure 1. It has a variable horizontal resolution grid with fixed 1.5 km resolution inner domain, stretching to 4 km at the outer boundary, and 80 vertical levels up to 40 km, with the lowest model level at 2.5 m. In this study, we focus on the Sydney domain, shown in blue (outer) and green (inner) in Figure 1 [14,15,16]. The variational assimilation system uses the observations and the background model short-range forecast from the previous cycle to generate an analysis increment. This increment is incorporated into the short-range forecast to generate the analysis, which is used as the initial forecast conditions for the next cycle of weather forecast products. The background forecast is at least long enough to span the assimilation window and produce the model background fields for the next assimilation cycle. A detailed description (e.g., observation processing and quality control) of the ACCESS-C assimilation system is found in [15].

### 2.2. The VarBC Configurations

VarBC is an adaptive bias correction method that updates the bias weights inside the data assimilation system. The observation bias is calculated at each new analysis cycle using the weights that were updated in the previous cycle and the current values of the predictors. The choice of predictors remains the same at each assimilation cycle. The predictors used in our data assimilation system are a scan position term (not part of the VarBC process but occasionally updated manually when instruments are recalibrated); a constant term, 850–300 hPa thickness, and 200–50 hPa thickness [5]; and four orthogonal Legendre polynomials that allow a more precise removal of scan bias [17]. The aim of VarBC is to minimize systematic innovations for satellite radiance observations and simultaneously improve the differences between the background and other observations in the assimilation system. This is done by finding the minimum value of the following cost function [10,17,18]:

(1) $$J_{VarBC}=J_{o}+J_{b}+\frac{{\left(\beta_{b}-\beta \right)}^{T}B_{\beta }^{-1}\left(\beta_{b}-\beta \right)}{2}$$

where *J_o_* minimizes the square error between the observations and analysis, *J_b_* minimizes the square error between the background and analysis, and third is the bias correction term, where bias parameters *β* are adjusted simultaneously with other analysis variables considering all available information to make an optimally unbiased analysis. The background coefficients *β_b_* were obtained from the previous analysis cycle. In this experiment, we used the initial coefficients *β_b_* from global model ACCESS-G when we started the assimilation experiment for the independent bias correction trial. *B_β_* is the background error covariance matrix for the bias coefficients (weights). This covariance matrix was diagonal with error variances of the background bias coefficients [10,17,18]:

(2) $$\sigma_{\beta_{b_{j}}}^{2}=\frac{\sigma_{o_{j}}^{2}}{N_{bgerr_{j}}}\;\left[j=1,\ldots \;\ldots ,N_{g}\times N_{p}\right]$$

where *σ_oj_* is the error standard deviation of the observed bias coefficient, and *N_bgerr_* is a user-defined positive integer parameter that determines the bias coefficient adaptivity rate in each analysis cycle. For simplicity and practical application, the same *N_bgerr_* is used for all predictors and all channels on each satellite instrument. At the Bureau, *N_bgerr_* is set to be [10,17,18]:

(3) Nbgerr=maxNavg,Nmin121n−1

where *N_avg_* is the expected number of the observations and is stored in the bias coefficients file and automatically updated at each data assimilation cycle, *N_min_* is a minimum number of observations to be used in updating the weights, and *n* is the bias-halving time. The *N_min_* and *n* are chosen by the user and can be varied to ensure that the coefficients do not adapt too quickly to transient spikes in the observation or model bias whilst adapting quickly enough that genuine changes in the instrument bias (such as due to the data provider changing the calibration parameters) are adjusted to quickly.

This VarBC scheme has been used in the Met Office global NWP system since 15 March 2016 after 7.5 months of extensive tests for satellite radiance observations [18] and in the Bureau model since 23 July 2019. Before VarBC, the models used a static bias correction scheme based on the method described in [5]. The current implementation only adjusts the bias of actively assimilated channels. The predictors used for most satellite-sounding instruments are the 850–300 hPa thickness and 200–50 hPa thickness layers of the atmosphere, along with scan position and a constant term.

We evaluated the performances of the VarBC-LAM and VarBC-global configurations in the framework of the limited-area model ACCESS-C over the Sydney domain. The ACCESS-G system runs operationally at the Bureau with a 6-h cycle 4 times per day (0000 UTC, 0600 UTC, 1200 UTC, and 1800 UTC). The same set of predictors is used in the ACCESS-G and ACCESS-C systems. In VarBC-global, the bias coefficients are updated from the global model ACCESS-G every 6 h at each global analysis time, without any initialization period. For the VarBC-LAM, we choose the following values for the controlling parameters: *N_min_* = 150 and *n* = 48. This means that, if there were to be at least 150 observations from a given satellite instrument channel in every assimilation cycle, a change in the bias would be halved in 48 h. In reality, the variable numbers of observations across different cycles means that the adaption rates will be slower. We used the *N_min_* chosen for operational use for the AMSU-B instrument in the Met Office United Kingdom variable resolution (UKV) limited-area convective scale model configuration since 11 July 2017. The Met Office uses lower values of *N_min_* for infrared sounders, for which some lower-peaking channels are not used frequently due to cloud contamination, in order to ensure a reasonable adaptation. However, we chose a smaller value of *n* than the Met Office (48 rather than 192; i.e., 2 days rather than 8 days), because our experience shows that biases adapt rather slowly in the Met Office and Bureau models in comparison with other centres. For our global model, we used a halving time of 1.5 days; the value chosen for our experiment was somewhat more conservative at 2 days. This halving time was shorter than that used by Benáček et al. [10], and we intend to perform more experiments to investigate the impact of changing the halving time in future experiments. However, we found little evidence that the bias corrections in VarBC-LAM are more variable cycle-to-cycle than VarBC-global.

### 2.3. Data and Experiment Design

Conventional observations assimilated in the ACCESS-C system are radiosondes; aircraft; and surface reports from ships, automatic weather stations (AWS), and buoys. Wind observations from derived from weather radar, satellite-derived atmospheric motion vectors (AMV), and surface winds from scatterometers are also used, along with lower tropospheric humidity-sensitive ground-based GNSS zenith total delay observations. In terms of satellite radiance observations, at the time of the study we used microwave radiances from the AMSU-B on polar satellites NOAA-19; Microwave Humidity Sounder (MHS) on MetOp-A, -B, and -C; Infrared Atmospheric Sounding Interferometer (IASI)on MetOp-A, -B, and -C; Advanced Technology Microwave Sounder (ATMS), and Cross-track Infrared Sounder (CrIS) on NOAA-20 and Suomi-NPP. At the time of the study, there was also occasional data assimilated from the Atmospheric Infrared Sounder (AIRS), but this has not been used since December 2020. MetOp-A was decommissioned in 2021.

The ACCESS-C model shares the same radiance processing as ACCESS-G, except that higher peaking channels, which are sensitivity to the atmospheric profile above the 40 km top of ACCESS-C, are not used. Additionally, the AMSU-A instrument is used in ACCESS-G coincident with the AMSU-B or MHS observations. Detailed information about the use of the observations and their quality control can be found in NOC Operations Bulletin Number 114, NOC Operations Bulletin Number 125, and BOC Operations Bulletin Number 105 [15,19,20,21,22,23]. The channels of AMSU-B/MHS, IASI, ATMS, and CrIS that we analysed for this study are listed in Table S1 (in the Supporting Information File). For AMSU-B/MHS and ATMS, these are the only channels assimilated. For CrIS and IASI, it is a standard subset used for monitoring at many NWP centres, including the Bureau. We assimilate 105 IASI channels and 65 CrIS channels into ACCESS-C. Spatial data thinning is undertaken in several stages; the first thinning is performed before data storage (see [24,25,26] for details), which preserves, for example, for IASI, 1 field of view out of 4. However, more restrictive thinning is undertaken after bias correction in ACCESS-C to ensure we have the most information possible for each observation before deciding which to keep. This later thinning is done with different spatial data lengths, depending on the observation. Only one observation is kept within each thinning data length. The data lengths are 24 km for AMSU-B/MHS, 60 km for IASI, 60 km for CrIS, 24 km for ATMS, and 60 km for AIRS. These settings were taken from our operational ACCESS-C model and were inherited from experiments performed at the Met Office.

We conducted two experiments in this study: Experiment 1 with VarBC-global (control) and Experiment 2 with VarBC-LAM (test), with details of the experiments shown in Table 1. For this study, we only analysed the observations over the ACCESS-C Sydney domain and ran assimilations from UTC-0000 1st February 2020 to UTC-2100 15th April 2020. Table 2 lists the satellite radiance observations assimilated into the ACCESS-C system and their availability over the SY domain at each cycle (averaged across one week).

## 3. Results and Discussion

**Figure 2:**
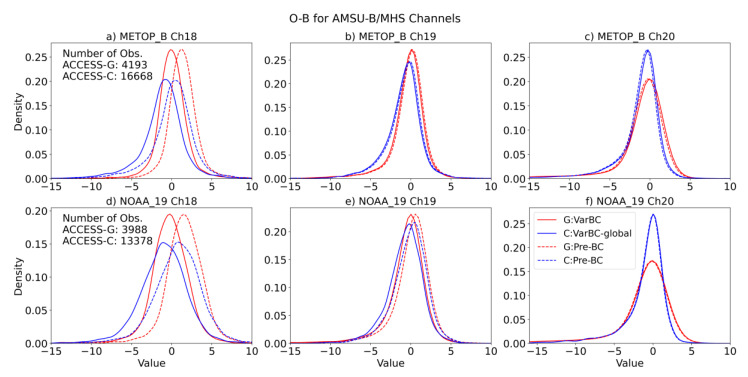
Density function of the observation-minus-background (O-B) differences for channels 18, 19, and 20 of MHS on MetOp-B (**a**–**c**) and AMSU-B on NOAA-19 (**d**–**f**) from Experiment 1. The differences are gathered separately for global ACCESS-G (red) and limited-area ACCESS-C (blue) when using the VarBC-global model set-up during Feb 2020. Dashed lines indicate the O-B of ACCESS-G and ACCESS-C without bias correction.

In the discussion of the results, we use the word background (or B) to refer to the model a priori state projected into the observation space through the use of the forward model operator used in the variational analysis system, which comprises an interpolation step to the observation location and a simulation using radiative transfer model RTTOV-11 (Radiative Transfer for TOVS-Version 11) [27]. The statistical distribution of observation minus simulated background (O-B) provides valuable diagnostic information to assess the performance of the data assimilation system and can be used to assess the effectiveness of the bias correction scheme to remove the bias between the observed and predicted model radiances. First, we computed the O-B (with bias correction and without bias correction) for the satellite instruments assimilated in Experiment 1 over a nearly one-month period (February 2020), with the same VarBC coefficients from the global model (i.e., ACCESS-G). Figure 2 and Figures S1–S3 show the O-B statistics for AMSU-B/MHS, IASI, CrIS, and ATMS.

In Figure 2, Figures S1 and S3, some channels show large biases (e.g., 18 in Figure 2; 73–108, 171, 196, and 201 in Figure S1; 27, 39, 47, 54, 61, 88, and 274 in Figure S2; and 22 in Figure S3). From the distribution of the O-B of all the instruments in both experiments, it can be seen that VarBC effectively removes biases when adopting the coefficients from the global model.

In the next experiment, we assimilated the same channels with the VarBC-LAM configuration (i.e., Experiment 2, independent bias correction applied in the LAM, not adopted from the global model, which means the bias coefficients update at each analysis time of ACCESS-C) over the same domain and period. Then, to evaluate the performance and impact of applying independent bias correction to ACCESS-C, we assessed the forecast skill using a range of tools developed by the UK Met Office [28,29]. It is usual, when implementing a change such as a new bias correction scheme or significant changes to the forward operator, new satellites, model changes, etc., to run the experiment for several weeks before beginning the evaluation period to allow the bias corrections to stabilize. In this case, we found no clear evidence that the bias corrections required a period of stabilization, although this analysis is limited by the general cycle-to-cycle variability in the average magnitude of the bias correction in LAMs. Furthermore, the close match in O-B distributions between the ACCESS-G and ACCESS-C models meant that the VarBC coefficients remained stable throughout the experiment period, and we evaluated the forecast performance over the full experiment period. This makes for a larger number of data points in the evaluation and more statistically robust results.

Due to the strong focus at the Bureau on precipitation forecast performance, we looked mainly at fraction skill scores (FSS) for precipitation forecasts (Roberts and Lean, 2007) verified against the Global Precipitation Measurement (GPM) data product (Hou et al., 2014) over the model domain. The FSS is determined by comparing forecasts with observations using fractional coverage over different-sized areas [23,30]. The FSS is preferred for precipitation verification in high-resolution NWP, because it rewards model skills in forecasting, for example, small-scale convective showers, even if their exact location and timing are not accurate. It provides a scale-aware analysis of rainfall compared with gridded rainfall products, and it is estimated for various rain rate thresholds and windows of N×N grid points.

**Figure 3:**
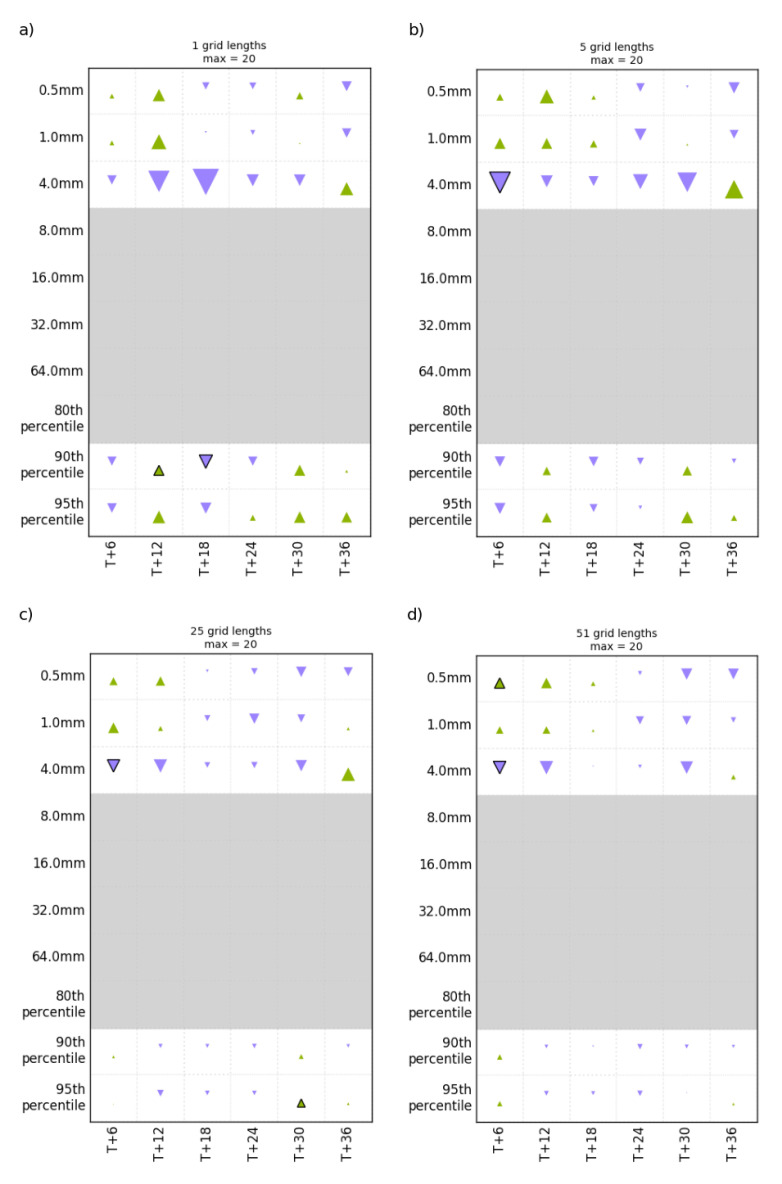
Hinton diagrams illustrating the differences in FSS for 1 h precipitation accumulation on a neighbourhood size of 1 (**a**), 5 (**b**), 25 (**c**), and 51 (**d**) grid lengths for the control and test. Green indicates a positive impact, and purple indicates a negative impact. Statistically significant results are triangles outlined in black.

Hinton diagrams [31] of the FSS performance of the Experiment 2 (test) model relative to Experiment 1 (control) at different grid lengths are shown in Figure 3. Hinton diagrams are used to visualize values of a two-dimensional (2D) array, ΔFSS, in which ΔFSS = FSS_Test_ − FSS_Control_ [32]. The positive and negative values in ΔFSS are represented by upward-pointing green and downward-pointing purple triangles, and the size of each triangle represents the magnitude of each value. The FSS statistics in Figure 3 indicate that the impacts are mixed when applying the independent bias correction in the LAM for all the window sizes. The degradation slightly increases (i.e., FSS from the control is higher) with the increase in window size and forecast length. Only a few results are statistically significant (marked with a bold outline), especially for shorter forecast lead times.

**Figure 4:**
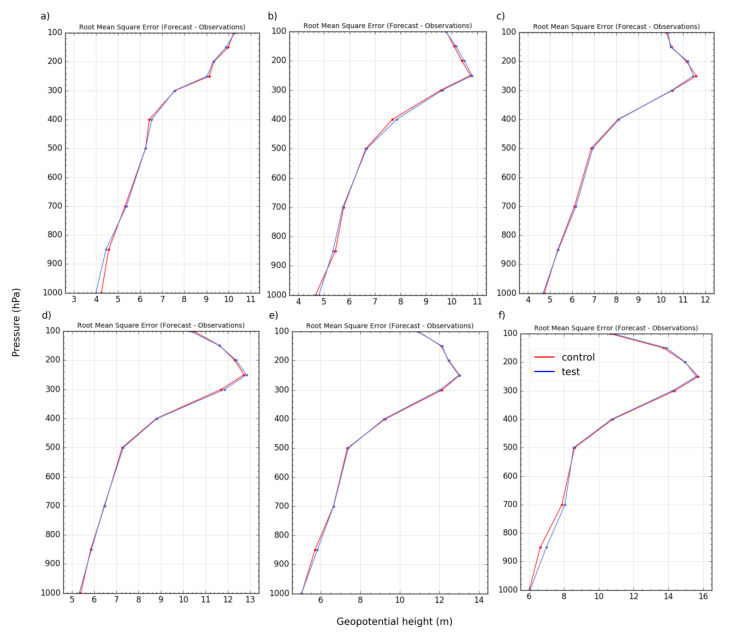
Vertical profile of the RMSE from the forecast at 6 h (**a**), 12 h (**b**), 18 h (**c**), 24 h (**d**), 30 h (**e**), and 36 h (**f**) for a geopotential height.

Satellite radiance observations have a more direct impact on the large-scale thermodynamics of the model than on the precipitation rates. The vertical profiles of the root mean square error (RMSE) of the forecast verified against radiosonde measurements for the geopotential height are also computed across the duration of the experiment (Figure 4). Examination of the RMSE shows that there is no significant difference between the control and test RMSE. Only a slight difference between them is noticeable around the 1000–700 hPa range. That indicates that the consequent impact on precipitation forecasts can be considered neutral.

**Figure 5:**
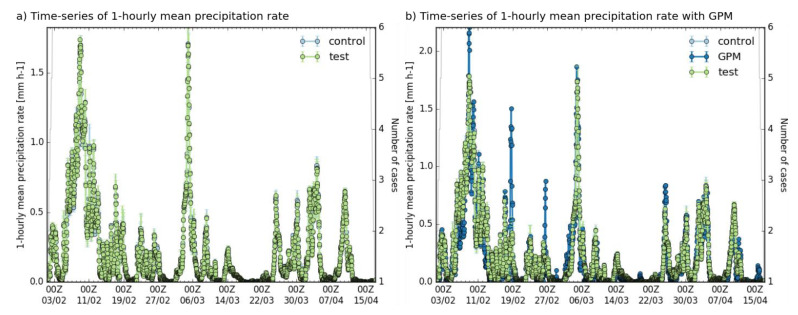
The temporal variations of the hourly mean precipitation rate between the control and test (**a**), and control, test and GPM (**b**).

Moreover, we computed the hourly mean precipitation rate between the control, test, and GPM (Figure 5). From Figure 5a, it is clearly seen that the hourly mean precipitation rate between the control and test is almost identical. Good agreement can also be seen in the time series of the precipitation rate between the GPM, control, and test (Figure 5b). However, a difference is apparent in the precipitation rate around the 9–10th, 19th, and 26–27th February; this may be because the precipitation intensity was relatively high on these days. The investigation of the spatial patterns of precipitation seen in each model run compared with GPM and radar data was inconclusive; neither the control nor test appeared to match the independent verification data particularly well for these cycles (not shown).

**Figure 6:**
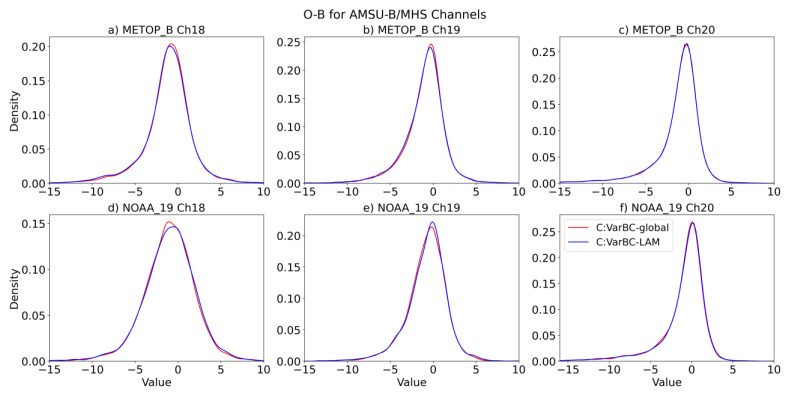
Density function of the O-B differences for the MHS (**a**–**c**) and AMSU-B (**d**–**f**) channels in ACCESS-C for the experiments VarBC-global (red) and VarBC-LAM (blue) during February 2020.

When making minor changes to the data assimilation, the impact can often be assessed more clearly by looking at the change in fit between the short-range forecast and radiance observations, which provides a lot of data points in a short period of time. We computed the O-B distribution (Figure 6 and Figures S4–S6, bias for the AMSU-B and ATMS channels assimilated, along with selected channels, to form IASI and CrIS, compared with Figure 2 and Figures S1–S3) and standard deviations in Table S2 in the Supporting Information File. These results suggest no significant difference in terms of independent bias correction for LAM compared to the adoption of global coefficients.

**Figure 7:**
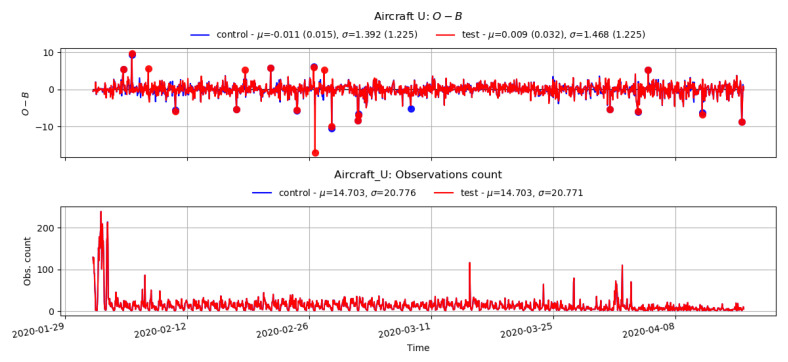
Time series plot of O-B (top), and the number of observations for Aircraft-U components from the control and test.

**Figure 8:**
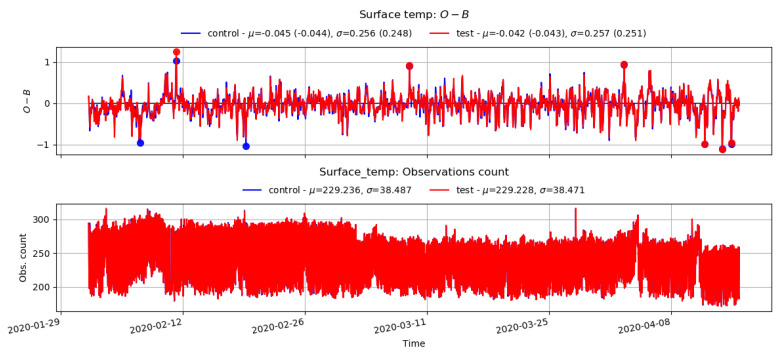
Time series plot of O-B (top), and the number of observations for the surface temperature from the control and test.

The VarBC scheme relies upon the DA system incorporating essential information from relatively unbiased anchor observations (e.g., radiosonde, aircraft, etc.) to ensure that the satellite data bias correction does not begin to reinforce the model climatological bias. Any such reinforcement of the model bias will lead to a drift in the bias when the model state is compared against anchor observations. Therefore, it is important to check whether applying VarBC in the assimilation system for satellite radiance observations will lead to a degradation in the model fit to the anchor observing systems. Figure 7 and Figure 8 show the time series of the 4D-Var (O-B) statistics (top plots) for Aircraft-U (longitudinal wind components) and the surface temperature from surface observations (automatic weather stations, ships, buoys, etc.) with the number of observations (bottom plot). Note that, for much of the trial period, the number of aircraft observations available was very low due to a technical issue and reduced further from mid-March due to the COVID-19 pandemic. Additionally, only a few radiosondes are available in our study, which generally report once daily but are not always available. Nevertheless, no significant difference was noticed between the control and test O-B statistics in both cases.

A spin-up (or initialization) period is not required for the VarBC-global method, since up-to-date bias coefficients are directly adopted from the global model to the LAM system at a certain regular assimilation cycle. The VarBC-LAM first requires a warm-up of the bias coefficients in the LAM conditions during the spin-up period. In this study, the VarBC-LAM does not start from zero bias coefficients (cold start), because we start from the relevant global model statistics (warm start). Hence, we anticipated minimal spin-up effects. Normally, we would monitor the size of the bias corrections and only verify the trial once the biases have stabilized, but in this case, since we found that the biases did not need to adapt much from the starting point, and the average bias corrections did not change significantly during the trial period; we used all available data in the analysis.

VarBC application in a LAM is not as straightforward as in a global model, and only a few studies have been conducted in this area [3,10]. To draw any more conclusions, the application of this scheme in the LAM domain needs further attention and extensive testing (over a more extended period and for different domains). Although the performance of independently applying VarBC in Experiment 2 showed the overall neutral impact in the current study, the experiment period was short, and any tendency to move towards reinforcement of the model bias due to inadequate anchor observations was unlikely to be seen in such a brief period on a small domain. Although the independent LAM VarBC configuration is similar to the global system, the VarBC adaptivity and predictors could be revised and set based on the meteorological conditions of the study area, NWP system performance, and radiance observations [32].

## 4. Conclusions and Proposed Further Study

This case study is dedicated to improving satellite data assimilation and radiance bias correction for the Bureau of Meteorology limited-area models. The difficulties of assimilating radiances from polar-orbiting instruments in limited-area model systems are well known. We applied independent bias correction for the limited-area model in this work. The O-B and forecast impact of the independent VarBC configuration were evaluated over two and half months. Overall, the verification statistics indicated little statistical difference between the control (VarBC-global) and test (VarBC-LAM) runs. Based on all the results, our investigation highlighted that the impact of using independent bias correction for the limited-area model is neutral.

There are advantages and disadvantages to running VarBC in the LAM. Given there is little difference in performance, it is perhaps safer to use the VarBC-global set-up, because this is statistically more robust due to the larger number of observations (both bias corrected and anchor). However, running VarBC has advantages for operational maintenance and would allow us to support the use of satellite radiances that are not assimilated in the global model. VarBC could be of particular benefit to the assimilation of high-resolution radiance observations from the Himawari-8 Advanced Himawari Imager (AHI), from which only the low-resolution Clear Sky Radiance (CSR) product is used in the global model. This early attempt to apply independent bias correction for the Bureau limited-area model will be built upon to try different strategies with respect to the predictor settings and to evaluate performance over an extended evaluation period and other seasons and regions of Australia. We also intend to incorporate GNSS-RO observations into our experiments to improve the bias characteristics of the models and, thus, the stability of the VarBC scheme.

## Figures and Tables

**Table 1:** Experiment details.

Item	Details
Experiment 1	VarBC-global (bias coefficient adopted from global model)
Experiment 2	VarBC-LAM (independent bias correction in LAM condition)
DA Method	4D-VAR
DA Cycle	Hourly (24)
Observations	Conventional: AWS, METAR reports, ships, buoys, radiosondes, aircraft, and AMV Nonconventional: GNSS zenith total delay; Scatterometer; radar; and radiances (ATMS, ATOVS, CrIS, and IASI)
ACCESS-C Model	Vertical Level: 80 Model top: 38.5 km Lowest model level: 2.5 m Grid Spacing: 1.5 km (inner, fixed) 4.0 km (outer, variable) No. of grid points: ~892 × 744
Trial period	1st Feb–15th April 2020

**Table 2:** Typical daily data availability per cycle over the SY domain in ACCESS-C.

Observations	Cycle (UTC)																							
	0	1	2	3	4	5	6	7	8	9	10	11	12	13	14	15	16	17	18	19	20	21	22	23
AIRS																								
IASI																								
CrIS																								
ATMS																								
AMSU-B/MHS																								

## Data Availability

All data generated or appeared in this study are available upon requested by contact with the corresponding author.

## Supplementary Materials

The following supporting information can be downloaded at: https://www.mdpi.com/article/10.3390/s22239504/s1, Figures S1–S6, Tables S1 and S2.
