# Supplementary material for: Testing Variational Bias Correction of Satellite Radiance Data in the ACCESS-C: Australian Convective-Scale NWP System
Source: Sensors (Basel). 2022 Dec 5;22(23):9504. doi: 10.3390/s22239504 (PMC9740059; doi:10.3390/s22239504)
Supplement: Supplementary file 1 [file sensors-22-09504-s001.zip › sensors-2033336-supplementary.pdf]

supplementary

Testing variational bias correction of satellite radiance data in the ACCESS-C: Australian Convective-Scale NWP system

Nahidul Hoque Samrat, Fiona Smith *, Jin Lee and Andrew Smith

The Bureau of Meteorology, Melbourne, VIC 3001, Australia
* Correspondence: fiona.smith@bom.gov.au

Introduction

This supporting information file contains the supplementary figures and tables referenced in the main article.

Table S1. Channels used for impact analysis in this study.

Instrument	Bureau channel number	Instrument Channel Number
AMSU-B/MHS	18	
	19	
	20	
IASI	73	242
	75	246
	85	280
	96	345
	98	350
	108	381
	122	434
	170	2239
	171	2245
	178	2889
	196	3029
	201	3058
CrIS	27	89
	28	93
	39	116
	45	130
	47	133
	54	145
	61	155
	71	166
	76	175
	88	248
	123	464
	274	854
	278	864
	297	921
ATMS	18	
	19	
	20	
	21	
	22	

Table S2. Standard deviations of the O-B of the channels for VarBC-global and VarBC-LAM.

**ATOVS : Metop_B**		
**Channel**	**VarBC-global**	VarBC-LAM
18	2.8	2.8
19	2.4	2.4
20	3.6	3.6
ATOVS : NOAA-19		
18	2.9	3.0
19	2.3	2.3
20	2.8	2.9
IASI		
73	0.3	0.3
75	0.3	0.3
85	0.3	0.4
96	0.3	0.3
97	0.4	0.4
98	0.5	0.5
108	0.5	0.5
122	0.5	0.5
170	1.3	1.3
171	1.0	1.0
178	1.6	1.6
196	1.9	1.9
201	1.7	1.7
CrIS		
27	0.2	0.2
28	0.2	0.2
39	0.2	0.2
45	0.2	0.2
47	0.2	0.2
54	0.3	0.3
61	0.5	0.5
71	0.5	0.5
76	0.8	0.8
88	1.0	1.0
123	1.1	1.1
274	1.5	1.5
ATMS		
18	9.1	9.1
19	7.0	7.1
20	5.4	5.5
21	7.0	7.1
22	3.6	3.6

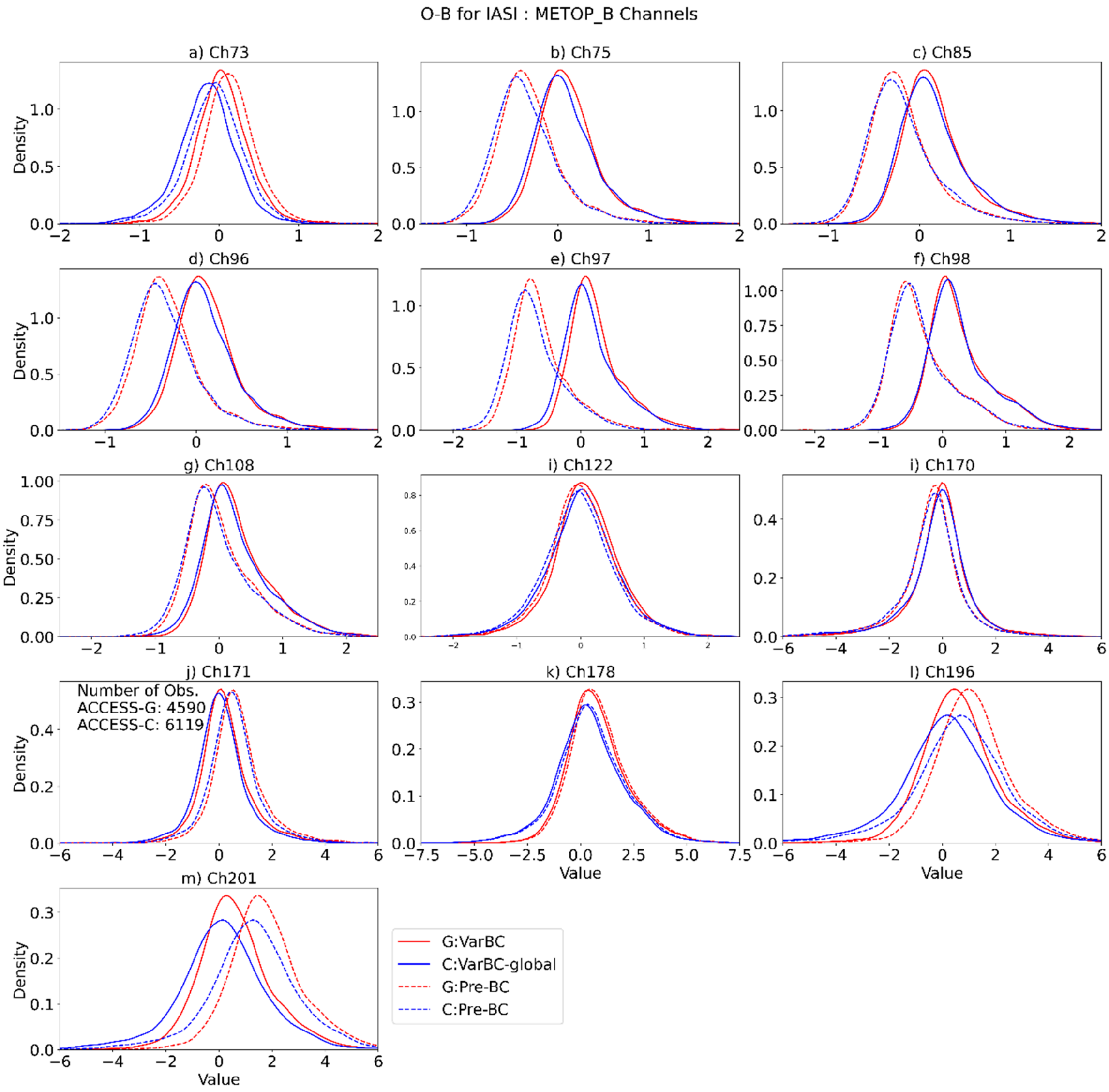

Figure S1. Density function of the observation-minus-background (O-B) differences for the IASI channels on MetOp-B from Experiment 1. The differences are gathered separately for global ACCESS-G (red) and limited-area ACCESS-C (blue) when using VarBC-global models during Feb 2020. Dashed line indicates the O-B of ACCESS-G and ACCESS-C without bias correction.

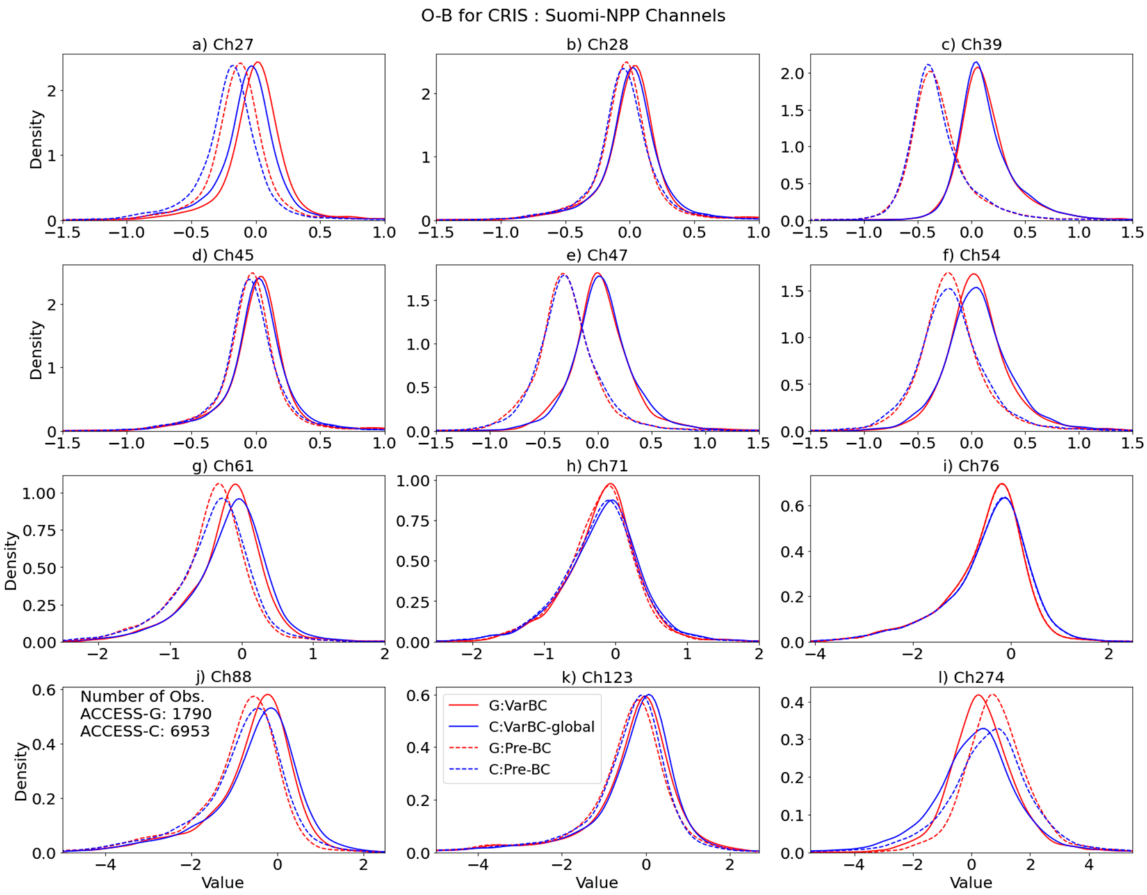

Figure S2. Density function of the observation-minus-background (O-B) differences for the CrIS channels on Soumi-NPP from Experiment 1. The differences are gathered separately for global ACCESS-G (red) and limited-area ACCESS-C (blue) when using VarBC-global models during Feb 2020. Dashed line indicates the O-B of ACCESS-G and ACCESS-C without bias correction.

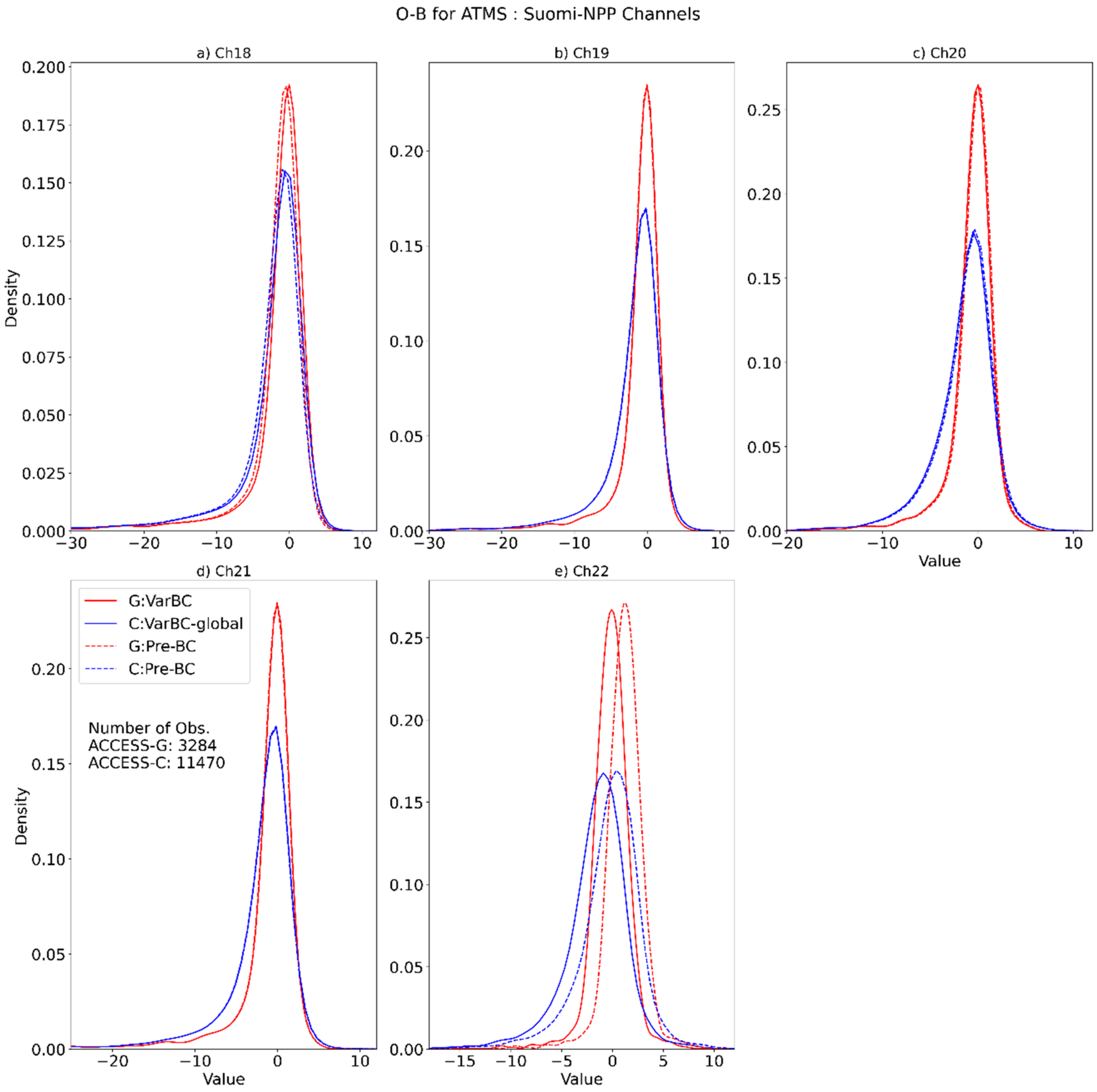

Figure S3. Density function of the observation-minus-background (O-B) differences for the ATMS channels on Suomi-NPP from Experiment 1. The differences are gathered separately for global ACCESS-G (red) and limited-area ACCESS-C (blue) when using VarBC-global models during Feb 2020. Dashed line indicates the O-B of ACCESS-G and ACCESS-C without bias correction.

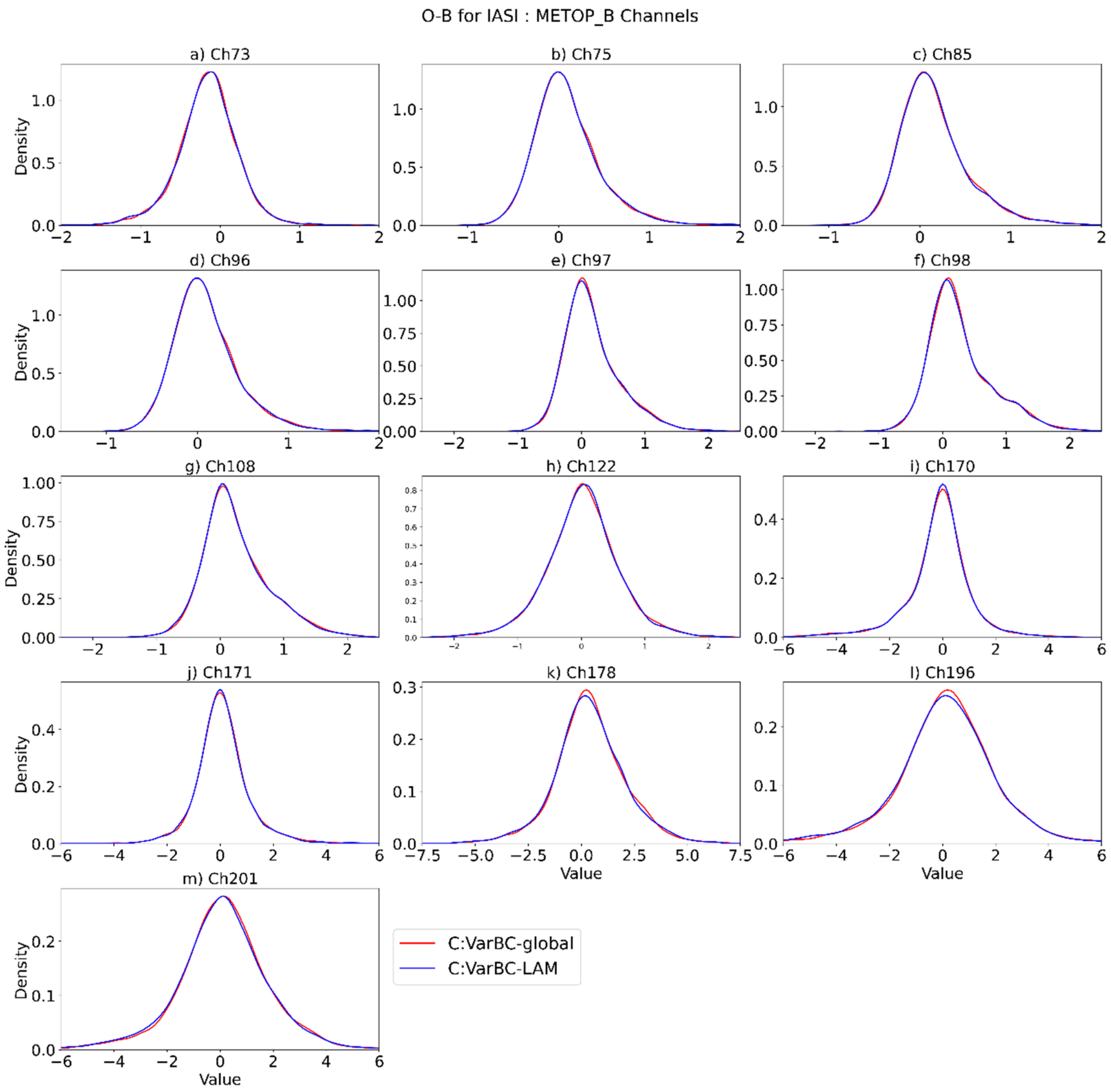

Figure S4. Density function of the O-B differences for the IASI channels. The differences are gathered separately for ACCESS-C when using the bias coefficient from the global model (red) and independent (blue) during Feb 2020.

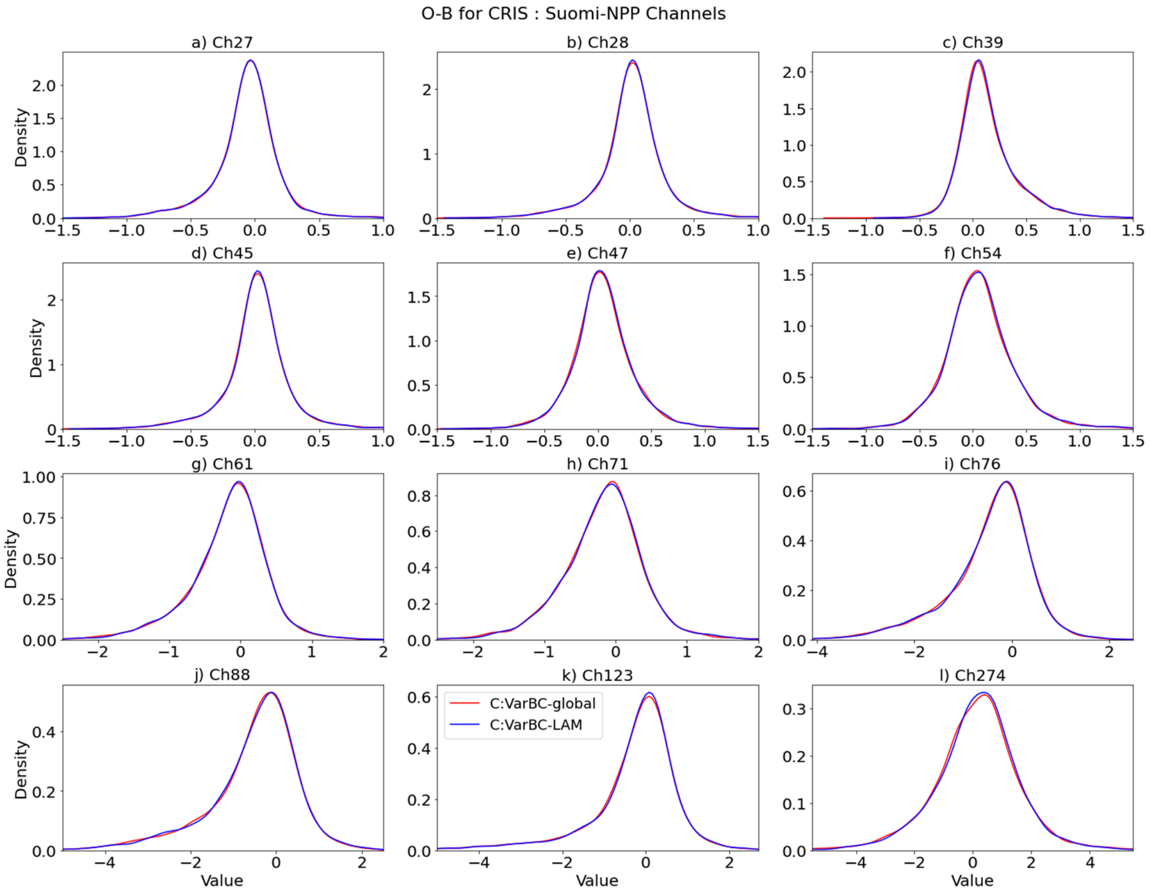

Figure S5. Density function of the O-B differences for the CrIS channels. The differences are gathered separately for ACCESS-C when using the bias coefficient from the global model (red) and independent (blue) during Feb 2020.

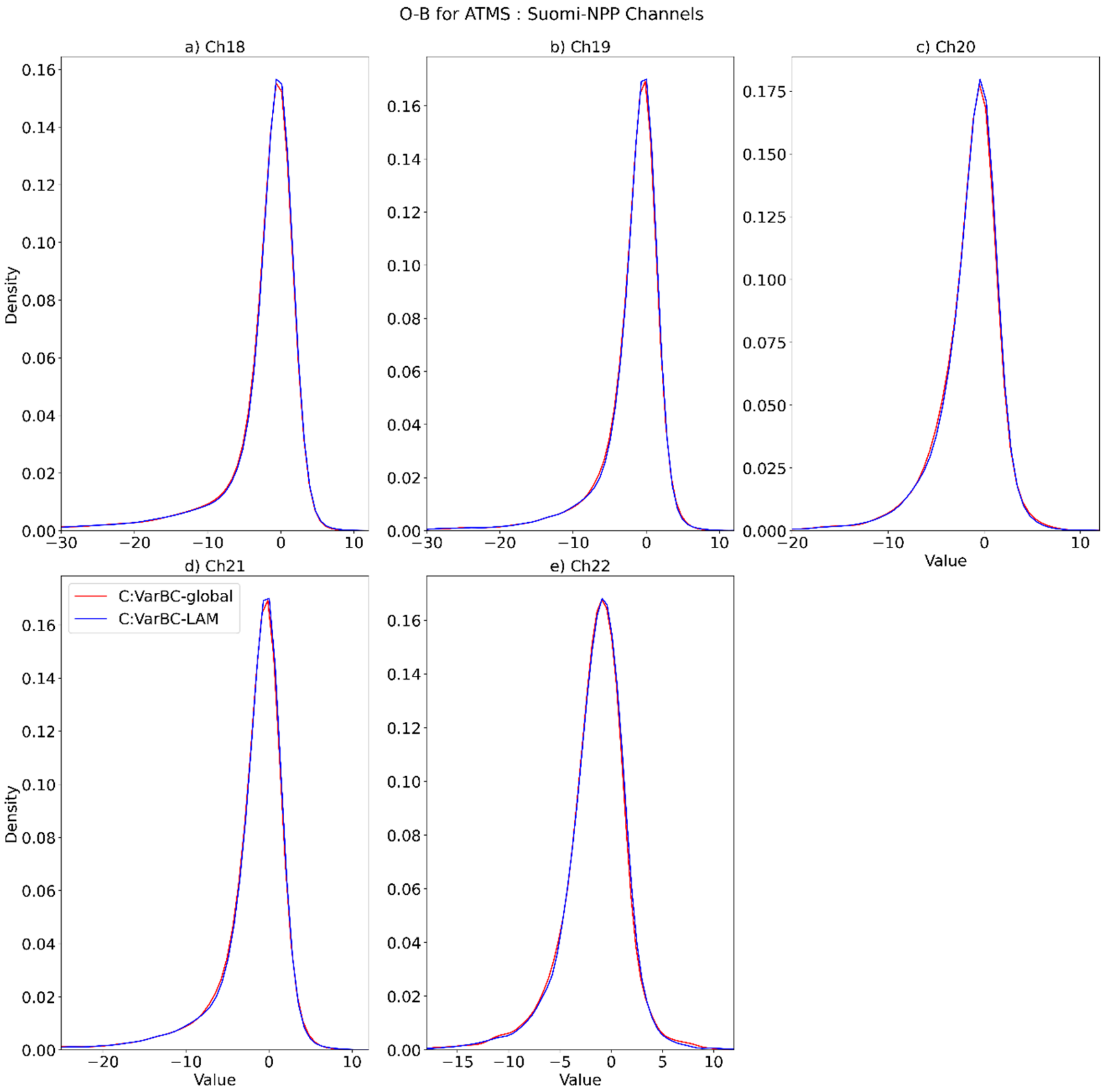

Figure S6. Density function of the O-B differences for the ATMS channels. The differences are gathered separately for ACCESS-C when using the bias coefficient from the global model (red) and independent (blue) during Feb 2020.
